# *Virgibacillus saliphilus* sp. nov. and *Virgibacillus salidurans* sp. nov., isolated from kimchi

**DOI:** 10.71150/jm.2501001

**Published:** 2025-01-24

**Authors:** Young Joon Oh, Joon Yong Kim, Min-Sung Kwon, Sulhee Lee, Sang-Pil Choi, Hak-Jong Choi

**Affiliations:** Technology Innovation Research Division, World Institute of Kimchi, Gwangju 61755, Republic of Korea

**Keywords:** *Virgibacillus*, *Virgibacillus salidurans*, *Virgibacillus saliphilus*, Kimchi, genome

## Abstract

This study aimed to provide a taxonomic description of two bacterial strains, NKC19-3^T^ and NKC19-16^T^, isolated from commercially produced kimchi obtained from various regions within the Republic of Korea. Both strains were rod-shaped, gram-stain-positive, facultatively anaerobic, and displayed positive reactions for oxidase and catalase. Additionally, these bacteria were motile, halophilic (salt-tolerant), and proliferated under alkaline conditions. Genetically, both strains showed 98.0% similarity in their 16S rRNA gene sequences and were most closely related to *Virgibacillus natechei* FarD^T^, with 96.5 and 96.8% sequence similarity, respectively. ANI values indicated that the two novel strains were distinct from *V. natechei* FarD^T^, as they were below the species demarcation threshold. The ANI value between strains NKC19-3ᵀ and NKC19-16ᵀ was 84.64–84.75%, and the values between these strains and other related strains did not exceed 80.0%, further supporting their classification as novel species. Phylogenetic analysis revealed that strains NKC19-3^T^ and NKC19-16^T^ formed a distinct branch within the genus *Virgibacillus*, clearly distinguishing them from other species in the same genus. Regarding genomic characteristics, the GC content was 38.9% for strain NKC19-3^T^ and 39.5% for strain NKC19-16^T^. The genome of strain NKC19-3^T^ had a size of approximately 4.1 Mb and contained 3,785 protein-coding genes (CDSs). Strain NKC19-16^T^ had a slightly smaller genome, approximately 3.9 Mb in size and harbored 3,726 CDSs. The polar lipid profiles of strains NKC19-3ᵀ and NKC19-16ᵀ included diphosphatidylglycerol (DPG), phosphatidylglycerol (PG), glycolipids (GL), and an unidentified lipid (L). The predominant fatty acids of both strains were anteiso-C_15:0_ and anteiso-C_17:0_. Considering the comprehensive analysis encompassing phenotypic, genomic, phylogenetic, and chemotaxonomic data, strains NKC19-3^T^ and NKC19-16^T^ are proposed to represent two novel species within the genus *Virgibacillus*. The suggested names for these species are *Virgibacillus saliphilus* sp. nov. (type strain NKC19-3^T^, also referred to as KACC 22326^T^ and DSM 112707^T^) and *Virgibacillus salidurans* sp. nov. (type strain NKC19-16^T^, also referred to as KACC 22327^T^ and DSM 112708^T^).

## Introduction

Heyndrickx et al. (1998) originally proposed the genus *Virgibacillus*. As of January 2024, this genus includes a total 35 species with a validly published and correct name. *Virgibacillus* species have been isolated from diverse sources, including traditional Korean salt-fermented foods such as salted seafood and kimchi (Kim et al., 2011; Oh et al., 2017), saline environments (An et al., 2007; Arahal et al., 1999, 2000; Carrasco et al., 2009; Wang et al., 2010; Yoon et al., 2004, 2010; Zhang et al., 2012), and soil (An et al., 2007; Carrasco et al., 2009). These bacteria typically exhibit gram-positive staining, rod-shaped cells, catalase-positive, halophilic preferences, and the ability to proliferate under aerobic or facultative anaerobic conditions. They typically contain menaquinone-7 (MK-7) as the major respiratory quinone, with their major fatty acids primarily being anteiso-C_15:0_ or iso-C_17:0_. The predominant polar lipid in *Virgibacillus* species is phosphatidylglycerol (PG), and their genomic GC content is within the range of 36 to 43% (Carrasco et al., 2009). Further details and updates regarding the genus *Virgibacillus* and its species can be found at the following link: https://www.ezbiocloud.net/search?tn=Virgibacillus. To date, 33 quality-controlled *Virgibacillus*-related genomes have been identified in the EzBioCloud public genome database. Based on these data, the median genomic properties of *Virgibacillus* are as follows: a total genome length of 4.2 Mb, a median number of protein-coding sequences (CDS) of 3,976, and a median GC content of 37.3%. In this study, we taxonomically characterize two novel strains, NKC19-3^T^ and NKC19-16^T^, isolated from kimchi. Through phylogenetic, phenotypic, and genomic analyses, we classified these strains as new members within the genus *Virgibacillus*.

## Materials and Methods

### Isolation of bacterial strains and culture conditions

To isolate the bacterial strains, two different commercially produced kimchi specimens were obtained from different regions in the Republic of Korea. Strain NKC19-3^T^ was isolated from baechu cabbage kimchi produced by a kimchi company located in Busan (35°14′40.2″ N 129°05′55.7″ E), while strain NKC19-16^T^ was isolated from baechu cabbage kimchi produced in Daegu (35°49′54.0″ N 128°37′39.5″ E) in 2019. The kimchi samples were chopped and filtered twice using sterilized gauze, after which serial kimchi soup dilutions were prepared and then spread onto halobacteria medium (M372; Deutsche Sammlung von Mikroorganismen und Zellkulturen [DSMZ] GmbH, Braunschweig, Germany) for cultivation. The M372 medium comprised 5 g yeast extract, 5 g casamino acids, 3 g sodium citrate, 1 g sodium glutamate, 20 g MgSO_4_∙7H_2_O, 2 g KCl, 36 mg FeCl_2_∙4H_2_O, 0.36 mg MnCl_2_∙4H_2_O, 200 g NaCl, and 20 g agar/L of distilled water. The bacterial plates were sealed with parafilm and incubated at 35°C for 2 weeks, preventing dehydration. The colonies of both strains observed on the M372 agar plates were subsequently subcultured several times to ensure their purity. The deposited strains, NKC19-3^T^ and NKC19-16^T^, were preserved at DSMZ and Korean Agricultural Culture Collection (KACC). Strain for taxonomic comparison, *V. natechei* FarD^T^ (DSM 25609^T^) was obtained from DSMZ. Routine culturing of the strains was performed on FarD medium (M1730) comprising 100 g NaCl, 10 g yeast extract, 7 g casamino acids, 3 g trisodium citrate, 5 g Na_2_CO_3_, 3 g KCl, 1 g MgSO_4_∙7H_2_O, and 20 g agar/L of distilled water. Incubation was conducted at 35°C for 5 days.

### Morphological and biochemical characterization

Morphology of strains NKC19-3^T^ and NKC19-16^T^ was examined using transmission electron microscopy (LIBRA 120; Carl Zeiss, Germany) and scanning electron microscopy (S-3500N; Hitachi, Japan). To assess their growth characteristics, both strains were cultured on M1730 medium at various temperatures (4, 10, 15, 20, 25, 30, 35, 37, 40, 45, 50, 55, and 60°C) and pH values (ranging from 4.0 to 11.0 with 1.0 pH unit intervals) for 5 days. The pH of the M1730 broth was adjusted using 100 mM 2-(N-morpholino) ethanesulfonic acid (MES) buffer for pH 4.0–6.0 and 100 mM N-Tris (hydroxymethyl) methyl-3-aminopropanesulfonic acid (TAPS) buffer for pH 7.0–11.0. After sterilization by autoclaving, the pH values were adjusted. The growth of both strains was also evaluated at different NaCl concentrations (0, 1, 2, 3, 5, 10, 15, 25, and 30%) on M1730 medium. To prepare the medium with varying NaCl concentrations, we sterilized NaCl separately and adjusted the concentrations accordingly during the preparation process. To determine optimal growth conditions, real-time cell growth loggers (RTS-1C; BioSan, Latvia) were used, as described by Oh et al. (2016). Anaerobic growth was assessed using an anaerobic chamber (Coy Laboratory Products Inc., USA) after 14 days of incubation at 35°C in an atmosphere composed of N_2_, CO_2_, and H_2_ (90:5:5 [v/v]) on M1730 agar with or without 50 mM NaNO_3_, 10 mM NaNO_2_, and 50 mM C_4_H_4_NaO_4_ (Lovley & Phillips, 1988). To evaluate motility, tests were conducted using M1730 medium containing 0.5% agar (Tittsler & Sandholzer, 1936). gram staining was performed using a BD gram staining Kit (BD Difco™; Becton Dickinson, USA), according to the manufacturer’s instructions. Microscopic evaluation was performed following gram staining and spore staining to observe the cellular and spore morphology of the strains. Oxidase and catalase tests were conducted using an oxidase reagent (bioMérieux, France) and 3% (v/v) H_2_O_2_, respectively. Endospore formation was observed using a method previously described by Salzman et al. (1993). Biochemical examinations of both strains were conducted using API 20NE and API ZYM kits (bioMérieux) following the manufacturer’s instructions with a minor modification that inoculation solution was adjusted to NaCl 10%. For additional phenotypic analyses, the API 50CH kit (bioMérieux, France) and Biolog GEN III microplates (Biolog, USA) were utilized according to the manufacturers' instructions, with a minor modification: the inoculation solution was adjusted to 10% (w/v) NaCl and a pH of 8.0. Tests for the hydrolysis of macromolecules were conducted by incubating all plates at 35°C for 5 days. Hydrolysis of Tween 60 and starch was tested by plating the two strains on an agar medium (15 g agar) containing 5 g peptone, 1 g yeast extract, 0.1 g K_2_HPO_4_, and either 1% (v/v) Tween 60 or 0.2% (w/v) starch. For starch hydrolysis, the plates were treated with Lugol’s iodine solution after incubation, and the formation of clearance zones was observed. Hydrolysis of DNA was determined using DNase Test Agar (Thermo Scientific) inoculated with the two strains. After incubation, the plates were treated with 1 M HCl to detect transparent zones around the colonies. Hydrolysis of casein was tested on an agar medium supplemented with 10% (w/v) skimmed milk powder inoculated with the two strains. Zones of clearance around the colonies were observed after incubation. Hydrolysis of cellulose was assessed by culturing the two strains on an agar medium containing 0.2% (w/v) cellulose and 0.5% (w/v) peptone. Following incubation, the plates were stained with 1% Congo red solution, washed with 1 M NaCl, and observed for zones of clearance or changes in color around the colonies.

### Phylogenetic analysis

To conduct phylogenetic analysis, genomic DNA extraction of strains NKC19-3^T^ and NKC19-16^T^ was performed using a Genomic DNA Prep Kit (SolGent, Korea). PCR was conducted to amplify the 16S rRNA genes of the isolates using the universal bacterial primers 27F and 1492R (Lane, 1991). The sequence assembly of the generated 16S rRNA gene fragments was carried out using the SeqMan program (DNAStar), and their similarity was confirmed using EzBioCloud BLAST (http://www.ezbiocloud.net) (Yoon et al., 2017). Sequences alignment was conducted using the RDP Aligner tool (https://rdp.cme.msu.edu). Phylogenetic trees were constructed using MEGA X software (Kumar et al., 2018) with the neighbor-joining (NJ) algorithms (Saitou & Nei, 1987), maximum-parsimony (MP) (Fitch, 1971), and minimum evolution (ME) method (Rzhetsky & Nei 1992). Kimura's two-parameter model was used to calculate genetic distances in the NJ and ME trees (Kimura, 1980), and bootstrap analysis was performed with 1,000 random iterations (Felsenstein, 1985). The Subtree-Pruning-Regrafting (SPR) algorithm was used to construct the MP tree (Nei & Kumar, 2000).

### Genome sequencing and annotation

The complete genomes of strains NKC19-3^T^ and NKC19-16^T^ were sequenced by DNALINK Inc. (Korea) using PacBio RSII (Pacific Biosciences, USA) sequencing technology. The obtained sequences were *de novo* assembled using RS HGAP version 3.0 software (Beck et al., 2016). The NCBI Prokaryotic Genome Annotation Pipeline (PGAP) was utilized to predict and annotate the genomes, following previously described methods (Tatusova et al., 2016). Additionally, functional annotation was conducted using the Kyoto Encyclopedia of Genes and Genomes (KEGG) databases (www.genome.jp/kegg) (Galperin et al., 2015; Huerta-Cepas et al., 2017), with eggNOG-mapper v. 2.1.12 (Cantalapiedra et al., 2021). Graphical circular genome maps of the novel strains were visualized using the EzBioCloud Whole Genome Analysis.

### Phylogenomic analysis and comparative genomic analysis

For downstream analyses, the reference sequences representing other *Virgibacillus* species were retrieved from the NCBI RefSeq database. All genomes were annotated with Prokka v. 1.14.6. Pan-genome analysis was conducted using BPGA v. 1.3 (Chaudhari et al., 2016). Default parameters were applied, and core, accessory, and unique genes were identified. Core genome alignment was performed with IQ-TREE v. 2.3.6, employing the GTR+G substitution model, along with 1,000 ultrafast bootstrap replicates and SH-aLRT tests for branch support. The resulting phylogenomic tree was visualized and annotated using the Interactive Tree of Life (iTOL) platform. To determine the distinctiveness of the strains from other *Virgibacillus* species, average nucleotide identity (ANI) values were calculated using the pyANI v. 0.2.12 (Pritchard et al., 2016). Subsequently, an ANI-based dendrogram of the genus *Virgibacillus* was constructed using UPGMA, following the methodology implemented in Python with the scipy library.

### Chemotaxonomic analysis

Isoprenoid quinones of strains NKC19-3^T^ and NKC19-16^T^ were analyzed using high-performance liquid chromatography (LC-20A; Shimadzu, Japan), following a previously described method (Hiraishi et al., 1998). To analyze cellular fatty acids, strains NKC19-3^T^ and NKC19-16^T^, along with the closely related strain, were cultured on M1730 medium at 35°C. The cellular fatty acids were extracted and analyzed using the Sherlock Microbial Identification System 6.0 (MIDI, TSBA6.0 database) method (Sasser, 1990). Two-dimensional thin-layer chromatography (TLC) was used to analyze the polar lipids of the two strains. Polar lipids were identified by spraying the TLC plates with specific reagents, including 5% ethanolic molybdophosphoric acid for total lipids, *α*-naphthol-sulfuric acid for glycolipids, ninhydrin for aminolipids, and molybdenum blue for phospholipids (Tindall et al., 1987).

### Accession numbers

The GenBank accession numbers for the 16S rRNA gene sequences and genomes of strains NKC19-3^T^ and NKC19-16^T^ are MW979941, MW979942, JAGYHC000000000, and CP074373, respectively.

## Results and Discussion

### Morphological, phenotypic, and biochemical characterization

Strains NKC19-3ᵀ and NKC19-16ᵀ exhibited similar characteristics, being facultatively anaerobic, motile, non-spore-forming, and rod-shaped bacteria. The strains were found to be gram-staining-positive and motile with peritrichous flagella (Fig. S1). After 5 days of growth on M1730, the cells of strain NKC19-3^T^ had a rod-shaped morphology with a width of 0.3–0.5 μm and a length of 1.8–2.2 μm, while those of strain NKC19-16^T^ were also rod-shaped with a width of 0.4–0.6 μm and a length of 1.6–1.9 μm (Fig. S1). The colonies of two strains were cream-like, opaque, circular, and approximately 0.8–1.2 mm in diameter. Optimal growth conditions for both strains were observed at a NaCl concentration of 10% (w/v), pH 8.0, and a temperature of 35°C. Strain NKC19-3ᵀ was capable of growth within a pH range of 5.0–9.0, at temperatures between 10°C and 45°C, and in NaCl concentrations of 2–25% (w/v). In contrast, strain NKC19-16ᵀ exhibited growth within a pH range of 6.0–10.0, at temperatures from 10°C to 40°C, and in NaCl concentrations of 2–20% (w/v). Both strains were able to grow anaerobically in M1730 medium after 2 weeks of incubation at 35°C. Growth was also observed when nitrate was added to the medium under the same conditions. Furthermore, strains NKC19-3^T^ and NKC19-16^T^ shared some common properties with the related type strain *V. natechei* FarD^T^, differing from it by showing positive results for the carbon utilization of glycerol and *N*-acetylglucosamine and for the enzyme activity of *α*-chymotrypsin and Naphthol-AS-BI-phosphohydrolase. Detailed morphological, phenotypic, and biochemical characteristics of strains NKC19-3^T^ and NKC19-16^T^ are shown in Table 1 and summarized in the species description.

### Phylogenetic analysis

**Fig. 1.**
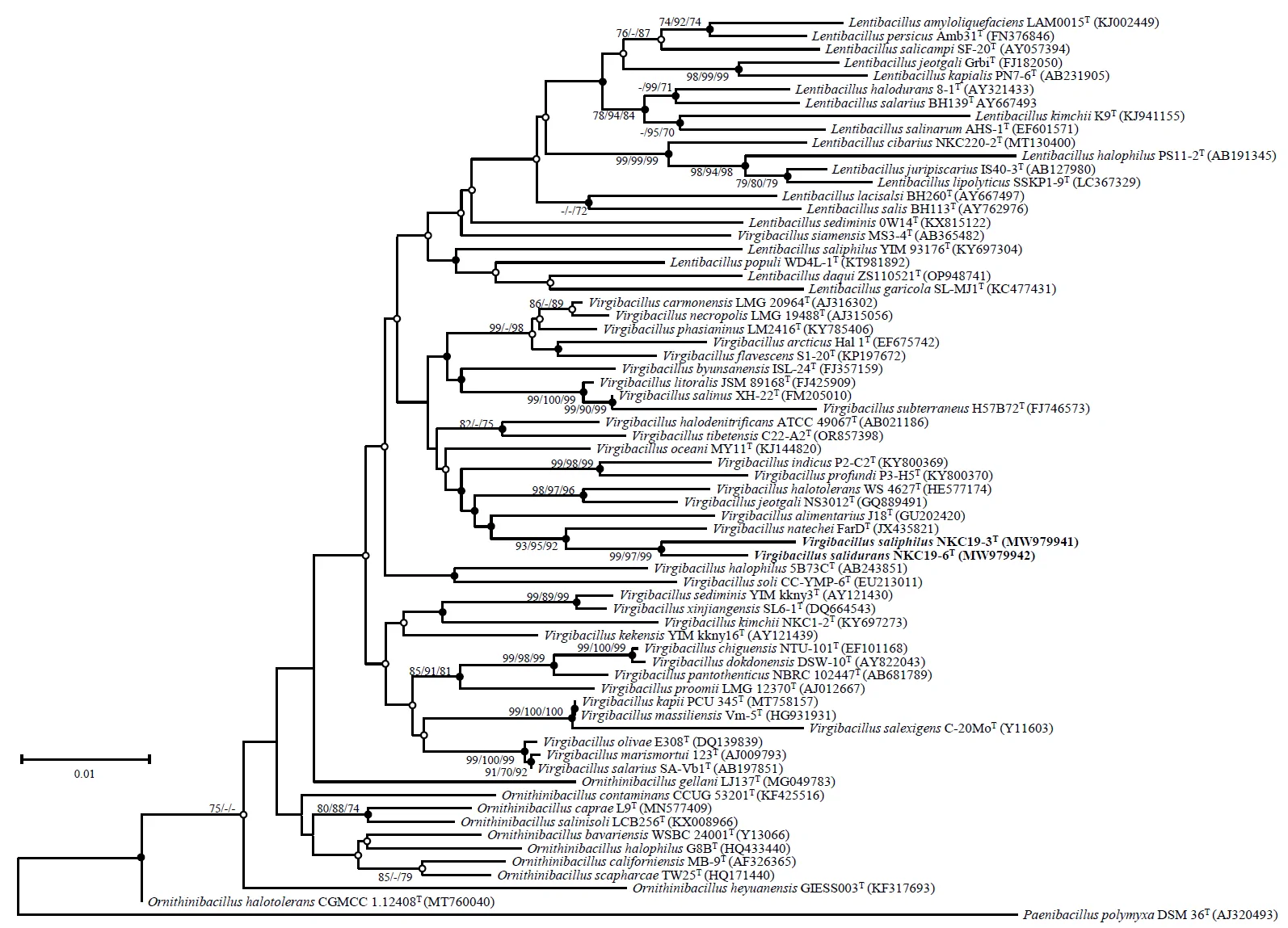
Phylogenetic tree based on neighbor-joining method showing the position of strains NKC19-3^T^ and NKC19-16^T^ among related taxa. Branch points by bootstrap values (> 70%) calculated by the neighbor-joining (NJ), maximum-parsimony (MP), and minimum-evolution (ME) methods. Filled circles indicate that the branch points were calculated using the MP and ME methods. Open circles indicate that the corresponding nodes were calculated using either the MP or the ME method. Scale bar represents 0.01 substitutions per nucleotide position.

The complete 16S rRNA gene sequences of strains NKC19-3^T^ and NKC19-16^T^ were determined through whole-genome sequencing analysis. The two strains exhibited a similarity ranging from 98.0% to 98.3%. Phylogenetic analysis based on the 16S rRNA gene sequences revealed that both strains were closely associated with *V. natechei* FarD^T^. Furthermore, they formed a distinct branch within the genus *Virgibacillus*, separate from other published species of the family *Bacillaceae* (Fig. 1).

### Genomic analysis

Strain NKC19-3^T^ had a single chromosome and one plasmid, with a total length of 4,054,365 bp and a GC content of 38.9%. It contained 3,785 coding sequences and 18 rRNA (six copies of 5S, six copies of 16S, and six copies of 23S), 5 ncRNA, and 65 tRNA genes. In comparison, strain NKC19-16^T^ had a single chromosome, with a length of 3,829,764 bp and a GC content of 39.5%. It contained 3,726 coding sequences and 18 rRNA (six copies of 5S, six copies of 16S, and six copies of 23S), 5 ncRNA, and 63 tRNA genes (Table S1 and Fig. S2).

The KEGG module analysis of strains NKC19-3^T^ and NKC19-16^T^ revealed that both strains possess a comprehensive set of complete metabolic pathways, supporting their survival and functionality in the kimchi fermentation environment (Table S2). In carbohydrate metabolism, both strains had complete pathways for glycolysis (M00001, M00002), gluconeogenesis (M00003), and the pentose phosphate pathway (M00004, M00006, M00007), ensuring efficient energy production and precursor generation for nucleotide biosynthesis. The TCA cycle is fully complete (M00009-M00011), enabling both strains to perform oxidative phosphorylation for maximal energy output. For energy metabolism, complete modules for respiratory pathways, including cytochrome bc1 complex (M00151), cytochrome c oxidase (M00155), and menaquinol oxidase (M00416), are present, along with ATP synthesis via F-type ATPase (M00157), highlighting robust energy-generating capabilities. In lipid metabolism, pathways for fatty acid biosynthesis initiation and elongation (M00082, M00083) and *β*-oxidation (M00086) are complete, indicating the ability to synthesize and degrade lipids for energy and structural components. The nucleotide metabolism pathways are fully functional, including *de novo* purine biosynthesis (M00048-M00050), pyrimidine biosynthesis (M00052), and deoxyribonucleotide synthesis (M00053), supporting DNA and RNA synthesis. Both strains also demonstrate complete amino acid metabolism, including biosynthesis pathways for threonine (M00018), methionine (M00017), cysteine (M00021), leucine (M00432), lysine (M00526), arginine (M00028, M00844), proline (M00015, M00970, M00972), and ectoine (M00033), alongside the glycine cleavage system (M00621). In cofactor and vitamin metabolism, both strains synthesize riboflavin (M00125), tetrahydrofolate (M00126), and heme (M00926), as well as pyridoxal phosphate (M00916), which are critical for enzymatic and redox activities. Additionally, C1-unit interconversion (M00140) and C10-C20 isoprenoid biosynthesis (M00364) pathways are complete, enabling essential metabolic flexibility and structural component synthesis.

While strains NKC19-3^T^ and NKC19-16^T^ shared extensive metabolic pathways, key differences highlight their functional specialization. Strain NKC19-3^T^ uniquely possesses complete modules for galactose degradation via the Leloir pathway (M00632) and UDP-galactose biosynthesis (M00554), indicating its ability to efficiently utilize galactose as an energy source. In contrast, strain NKC19-16^T^ demonstrates enhanced environmental adaptability and unique metabolite production, with complete modules for formaldehyde assimilation (M00345) and siroheme biosynthesis (M00846), enabling detoxification and nitrate/sulfate reduction. Furthermore, strain NKC19-16^T^ completes pathways for lysine biosynthesis via the DAP aminotransferase pathway (M00527), GABA biosynthesis (M00135), and histidine degradation (M00045), suggesting its role in producing functional metabolites that may contribute to flavor development and stress tolerance during fermentation. These findings suggest that strain NKC19-3^T^ specializes in carbohydrate utilization, while strain NKC19-16^T^ excels in environmental adaptation and functional metabolite synthesis, highlighting their complementary roles in the kimchi fermentation process.

### Comparative genomic analysis

The ANI value between strains NKC19-3^T^ and NKC19-16^T^ was 84.64–84.75%, and the values between these strains and other related strains did not exceed 80.0%, indicating their distinctiveness from other species within the genus *Virgibacillus* (Fig. S3).

Pan-genomic analysis exhibited that NKC19-3^T^ and NKC19-16^T^ and the 26 related strains had 81,853 pan-genomes. In addition, 910 core genomes were present in all strains; strains NKC19-3^T^ and NKC19-16^T^ had 597 and 347 strain-specific unique genomes, respectively.

The phylogenomic analysis revealed that the two novel strains, NKC19-3^T^ and NKC19-16^T^, were closely related and form a distinct clade within the *Virgibacillus* genus (Fig. S4). These strains clustered with *Virgibacillus natechei* and exhibited high genetic similarity, indicating a shared evolutionary lineage. However, the branch lengths separating NKC19-3^T^ and NKC19-16^T^ from *V. natechei* suggest that they have undergone genetic divergence, potentially reflecting distinct ecological adaptations or unique genomic features. The findings position NKC19-3^T^ and NKC19-16^T^ as novel members within the *Virgibacillus* genus, contributing to the understanding of phylogenetic relationships and genetic diversity within this group. Further analysis of their genomic and functional characteristics will provide valuable insights into their ecological roles and potential applications.

### Chemotaxonomic characteristics

The predominant isoprenoid quinone of strains NKC19-3^T^ and NKC19-16^T^ was MK-7, which is characteristic of the genus *Virgibacillus*. The major cellular fatty acids of both strains were anteiso-C_15:0_ (59.3%, 64.4%) and anteiso-C_17:0_ (34.1%, 13.8%), consistent with those of *V. natechei* DSM25609^T^ (88.3%, 5.4%). Detailed characteristics are presented in the subsequent species description and Table 2. The polar lipids of strain NKC19-3^T^ comprised diphosphatidylglycerol (DPG), phosphatidylglycerol (PG), two unidentified aminophospholipids (APL), an unidentified glycolipid (GL), two unidentified phospholipids (PL), and an unidentified lipid (L). The polar lipids of strain NKC19-16^T^ comprised DPG, PG, two GLs, L, and an aminolipid (AL). The polar lipid profiles of both strains contained GL and L, differing from those of the closely related strain (Fig. S5). Similar to other *Virgibacillus* species, strain NKC19-16ᵀ contained DPG and PG as major polar lipids. Unlike *Lentibacillus* species, the predominant fatty acids were identified as anteiso-C_15:0_ and anteiso-C_17:0_. The distinguishing characteristics of this strain compared to other species are presented in Table 1.

Taken together, the phenotypic, genomic, phylogenetic, and chemotaxonomic results suggest that NKC19-3^T^ and NKC19-16^T^ may represent two novel species in the genus *Virgibacillus*, for which the names *Virgibacillus saliphilus* sp. nov. and *Virgibacillus salidurans* sp. nov. are proposed, with NKC19-3^T^ and NKC19-16^T^ as type strains, respectively.

### Description of *Virgibacillus saliphilus* sp. nov.

*V. saliphilus* (sa. li’phi.la. L. masc. n. /sal, salis salt; Gr. masc. adj. philos loving; N.L. masc. adj. saliphilus salt-loving).

Cells were gram-positive, motile, facultatively anaerobic, and non-spore-forming rods that were 0.3–0.5 μm in width and 1.8–2.2 μm at length. Colonies were cream-colored, circular, convex, smooth, and 0.8–1.2 mm in diameter after 5 days on M1730 at 35°C. Growth occurred in 2–25% (w/v) NaCl (optimal in 10%) and pH 5.0–9.0 (optimal at pH 8.0) at 10–45°C (optimal at 35°C). The isolate was positive for catalase and oxidase. Positive for the hydrolysis of starch, DNase, tween-20 and tween-60 but not casein and cellulose. Using API 20NE (bioMérieux), strain was nitrate reduction, glucose fermentation, esculin, gelatin, and PNPG and negative for indole, arginine dihydrolase, and urease. When assayed with the API ZYM system, cells were positive for esterase (C4), esterase lipase (C8), acid phosphatase, alkaline phosphatase, *α*-chymotrypsin, *β*-galactosidase, and naphthol-AS-BI-phosphohydrolase and negative for cysteine arylamidase, leucine arylamidase, valine arylamidase, lipase (C14), trypsin, *α*-galactosidase, *β*-glucuronidase, *α*-glucosidase, *β*-glucosidase, *α*-mannosidase, *α*-fucosidase, and *N*-acetyl-*β*-glucosaminidase. Acid was produced from D-glucose, D-ribose, glycerol, D-galactose, D-fructose, D-mannose, *N*-acetylglucosamine, arbutin, esculin, salicin, D-lactose, D-melibiose, gentiobiose, D-tagatose, and 5-ketogluconate (API 50CH tests). Utilization of D-cellobiose, D-maltose, D-trehalose, D-melibiose, gentiobiose, *α*-D-lactose, D-turanose, D-salicin, sucrose, stachyose, *β*-methyl-D-glucoside, *N*-acetyl-*β*-D-mannosamin, *N*-acetyl-D-glucosamine, *N*-acetyl neuraminic acid, *N*-acetyl-D-galactosamine, D-fructose, *α*-D-glucose, 3-methyl glucose, D-mannose, D-galactose, D-sorbitol, inosine, D-fucose, L-fucose, D-mannitol, D-arabitol, myo-inositol, glycerol, L-rhamnose, D-glucose-6-phosphate, D-fructose-6-phosphate, D-aspartic acid, D-serine, L-alanine, L-arginine, glycyl-L-proline, L-aspartic acid, L-glutamic acid, L-histidine, L-pyroglutamic acid, L-serine, D-glucuronic acid, D-gluconic acid, L-galactonic acid, D-galacturonic acid, lactone, glucuronamide, mucic acid, D-saccharic acid, quinic acid, *p*-hydroxyphenylacetic acid, methyl pyruvate, L-lactic acid, D-lactic acid methyl ester, *α*-keto-glutaric acid, citric acid, D-malic acid, L-malic acid, γ-amino-butyric acid, bromo-succinic acid, *α*-keto-butyric acid, *α*-hydroxy butyric acid, *β*-hydroxy-D,L-butyric acid, acetoacetic acid, propionic acid, acetic acid, Tween 40, and formic acid was positive, and negative for dextrin, D-raffinose, gelatin, and pectin. The major cellular fatty acids were anteiso-C_15:0_ and anteiso-C_17:0_. The major polar lipids were diphosphatidylglycerol (DPG), phosphatidylglycerol (PG), two unidentified aminophospholipids (APLs), an unidentified glycolipid (GL), two unidentified phospholipids (PLs), and an unidentified L. The isoprenoid quinone was MK-7. The genomic DNA GC content of the novel strain was 38.9%.

The type strain, NKC19-3^T^ (=KACC 22326^T^, =DSM 112707^T^), was isolated from kimchi, a traditional fermented food from the Republic of Korea.

### Description of *Virgibacillus salidurans* sp. nov.

*Virgibacillus salidurans* (sa.li.du’rans. L. masc. n. sal salis, salt; L. pres. part. durans resisting; N.L. part. adj. salidurans, salt-resisting).

Cells were gram-positive, motile, non-spore-forming, and facultative anaerobic rods 0.4–0.6 μm in width and 1.6–1.9 μm in length. Growth occurred in 2–20% (w/v) NaCl (optimal in 10%) and pH 6.0–10.0 (optimal at pH 8.0) at 10–40°C (optimal at 35°C). The isolate was positive for catalase and oxidase. It was positivie for the hydrolysis of starch, and DNase but not casein, tween-20, tween-60 and cellulose. Using API 20NE (bioMérieux), strain was nitrate reduction, glucose fermentation, esculin, and gelatin and negative for indole, arginine dihydrolase, urease, and PNPG. When assayed with the API ZYM system, cells were positive for esterase (C4), esterase lipase (C8), leucine arylamidase, valine arylamidase, alkaline phosphatase, *α*-chymotrypsin, acid phosphatase, *N*-acetyl-*β*-glucosaminidase, and naphthol-AS-BI-phosphohydrolase and negative for cysteine arylamidase, trypsin, lipase (C14), *β*-glucuronidase, *β*-galactosidase, *α*-galactosidase, *α*-glucosidase, *β*-glucosidase, *α*-fucosidase, and *α*-mannosidase. Acid was produced from D-ribose, D-xylose, glycerol, D-glucose, D-fructose, L-sorbose, D-mannitol, D-sorbitol, esculin, *N*-acetylglucosamine, D-trehalose, D-tagatose, xylitol, D-arabinose, L-arabinose, D-arabitol, gluconate, 2-ketogluconate, and 5-ketogluconate (API 50CH tests). Utilization of D-maltose, D-trehalose, D-cellobiose, gentiobiose, dextrin, sucrose, stachyose, D-turanose, D-melibiose, D-raffinose, *N*-acetyl-D-glucosamine, *N*-acetyl-*β*-D-mannosamin, *N*-acetyl-D-galactosamine, D-fructose, D-mannose, D-galactose, D-mannitol, D-fucose, L-rhamnose, L-fucose, D-sorbitol, D-arabitol, myo-inositol, glycerol, D-glucose-6-phosphate, D-serine, L-alanine, L-glutamic acid, L-histidine, citric acid, D-lactic acid methyl ester, L-malic acid, *α*-keto-glutaric acid, *β*-hydroxy-D,L-butyric acid, Tween 40, acetoacetic acid, propionic acid, and acetic acid was positive and negative for D-salicin, *α*-D-lactose, *β*-methyl-D-glucoside, *α*-D-glucose, 3-methyl glucose, *N*-acetyl neuraminic acid, inosine, D-fructose-6-phosphate, gelatin, D-aspartic acid, glycyl-L-proline, L-arginine, L-aspartic acid, L-pyroglutamic acid, L-serine, D-galacturonic acid, D-gluconic acid, L-galactonic acid lactone, D-glucuronic acid, pectin, mucic acid, glucuronamide, D-saccharic acid, quinic acid, *p*-hydroxyphenylacetic acid, methyl pyruvate, L-lactic acid, D-malic acid, bromo-succinic acid, γ-amino-butryric acid, *α*-hydroxy butyric acid, *α*-keto-butyric acid, and formic acid. The major cellular fatty acids were anteiso-C_15:0_ and anteiso-C_17:0_. The major polar lipids were diphosphatidylglycerol (DPG), phosphatidylglycerol (PG), two unidentified glycolipids (GLs), an aminolipid (AL), and an unidentified lipid (L). The isoprenoid quinone was MK-7. The genomic DNA GC content of the novel strain was 39.5%.

The type strain, NKC19-16^T^ (=KACC 22327^T^, =DSM 112708^T^), was isolated from kimchi, a traditional fermented food from the Republic of Korea.

## Figures and Tables

**Table 1.** Differential characteristics of stains NKC19-3^T^, NKC19-16^T^, and relative taxa Taxa: 1, NKC19-3^T^, 2, NKC19-16^T^, 3, *Virgibacillus natechei* DSM25609^T^, 4, *Virgibacillus salinus* XH-22^T^ (data from [Carrasco et al., 2009]), 5, *Virgibacillus alimentarius* J18^T^ (Kim et al., 2011), 6, *Virgibacillus halotolerans* WS 4627^T^ (Seiler & Wenning, 2013), 7, *Virgibacillus phasianinus* LM2416^T^ (Tak et al., 2018), 8, *Virgibacillus oceani* MY11^T^ (Yin et al., 2015), 9, *Virgibacillus necropolis* LMG 19488^T^ (Heyrman et al., 2003), 10, *Virgibacillus halodenitrificans* DSM 10037^T^ (Yoon et al., 2004), 11, *Virgibacillus profundi* P3-H5^T^ (Xu et al., 2018), 12, *Virgibacillus pantothenticus* LMG 7129^T^ (Heyndrickx et al., 1998), 13, *Lentibacillus sediminis* 0W14^T^ (Guo et al., 2017), 14, *Lentibacillus salicampi* SF-20^T^ (Yoon et al., 2002). All taxa contain anteiso C_15.0_ as a major fatty acid.

	1	2	3	4	5	6	7	8	9	10	11	12	13	14
**Endospore formation**	-	-	+	+	+	+	ND	+	+	+	+	+	+	+
**Gram stain**	+	+	+	+	+	+	+	+	+	V	V	+	+	v
**Motility**	+	+	+	+	+	w	+	+	+	+	+	+	+	+
**Anaerobic growth**	+	+	+	-	-	-	-	-	-	+	+	+	+	-
**NaCl range**	2–25%	2–20%	1–20%	3–20%	0–30%	0.5–16.5%	0–20%	0.5–18%	0–10%	0.5–25%	4–30%	0.5–20%	1–20%	2–23%
(optimum, w/v)	(10%)	(10%)	(10%)^a^	(10%)	(9–10%)	(3–5%)	(10%)	(3.5%)	(5–10%)	(2–6%)	(15–25%)	(4–10%)	(6%)	(4–8%)
**Temperature range**	10–45	10–40	10–40^a^	4–40	4–40	8–35	10–30	15–45	10–40	4–42	0.5–15	10–50	15–45	15–40
(optimum, °C)	(35)	(35)	(35)^a^	(35-37)	(37)	(27–30)	(30)	(35–37)	(25–35)	(35)	(3–7)	(37)	(37)	(30)
**pH range**	5–9	6–10	6–12^a^	6–10	7–11	6.5–8.5	6–7	6–10	ND	6.0–9.5	6.5–9.0	ND	7–9.5	ND
(optimum)	(8)	(8)	(7)^a^	(7.5)	(10)	(7–8)	(6–7)	(8–9)	ND	(7.0–7.5)	ND	(7)	(7.5)	(6–8)
**Catalase**	+	+	+	+	-	+	+	+	+	+	+	+	+	+
**Oxidase**	+	+	+	-	-	+	-	+	+	+	+	-	-	+
**Nitrate reduction**	+	+	+	+	ND	-	+	+	+	+	+	+	+	+
**Hydrolyses of :**														
Casein	-	-	-	+	-	+	ND	+	-	-	+	-	ND	+
Starch	w	+	-	ND	-	-	ND	-	+	-	-	+	-	-
**Acid production from:**														
D-Galactose	+	-	-	+	-	+	+	w	-	+	w	-	+	-
D-Glucose	+	+	+	+	-	+	+	w	+	+	+	-	+	-
D-Fructose	+	+	-	+	-	+	+	+	+	v	+	-	+	-
D-Mannitol	-	+	+	-	-	-	+	-	-	+	w	-	+	-
D-Rhamnose	-	-	+	+	-	+	+	-	-	-	-	+	-	-
Trehalose	-	+	-	+	-	+	+	w	W	+	-	+	+	-
**Major fatty acids**	C_17:0_	C_17:0_	C_17:0_	C_16:0_	C_17:0_	C_17:0_	C_17:0_	C_17:0_	C_17:0_	C_17:0_	C_17:0_	C_17:0_	C_15:0_ iso	C_16:0_ iso
	anteiso	anteiso	anteiso		anteiso	anteiso	anteiso	anteiso	anteiso	anteiso	anteiso	anteiso		
**Polar lipids^b^**	DPG, PG, APLs, GL, PLs, L	DPG, PG, GLs, AL, L	DPG, PG, PLs, Ls	DPG, PG, GL, PLs	DPG, PG, PE, Ls	DPG, PG	PG, DPG, PLs, APLs	DPG, PG, Ls, PLs	PG, DPG, PE, PLs	PG, DPG	PG, DPG	ND	DPG, PG, GLs, PLs	DPG, PG, Ls
**G + C content (mol%)**	38.9^c^	39.5^c^	38.4^c^	38.8	37.1	39.1	39.5	34.2	37.3	38.0–39.0	37.3	36.9–38.3	44.8	44

All data were obtained in this study unless stated otherwise. Symbols: +, positive reaction; -, negative reaction.

a Data from Amziane et al. (2013).

b DPG, diphosphatidylglycerol; PG, phosphatidylglycerol; APL, unidentified aminophospholipid; GL, unidentified glycolipid; PL, unidentified phospholipid; L, unidentified lipid.

c DNA G + C content of strains was calculated from the respective genome.

**Table 2.** The cellular fatty acid compositions of strain NKC19-3^T^, NKC19-16^T^, and related taxa Taxa: 1, NKC19-3^T^; 2, NKC19-16^T^; 3, *Virgibacillus natechei* DSM25609^T^. All data were obtained in this study. The major fatty acids (>20% of the total) are indicated in bold. ND, not detected.

Fatty acids	1	2	3		
**Saturated**					
C_10:0_	ND	0.14	ND		
C_14:0_	0.34	0.82	0.49		
C_16:0_	1.18	2.09	1.11		
**Unsaturated**					
C_16:1_ ω7c alcohol	0.10	0.21	ND		
**Branched **					
C_14:0_ iso	0.50	4.15	1.86		
C_15:0_ iso	0.92	4.56	1.07		
C_16:0_ iso	2.42	8.74	1.18		
C_17:0_ iso	0.30	0.72	ND		
C_13:0_ anteiso	0.25	0.43	0.54		
C_15:0_ anteiso	**59.31**	**64.39**	**88.34**		
C_17:0_ anteiso	**34.10**	13.76	5.42		
Summed feature					
4; C_17:1_ iso / C_17:1_ anteiso B	0.57	ND	ND		

## Supplementary Information

Table S1. Genomic features of strains NKC19-3^T^, NKC19-16^T^, and DSM25609^T^. All data were from this study unless stated otherwise

Table S2. KEGG Module comparison between strain NKC19-3^T^ and strain NKC19-16^T^

Table S3. Differential characteristics of stains NKC19-3^T^, NKC19-16^T^, and relative taxa

Fig. S1. Transmission electron micrographs and scanning electron micrographs of strains NKC19-3^T^ (A, B) and NKC19-16^T^ (C, D) grown in M1730 medium supplemented with 10% NaCl for 5 days.

Fig. S2. Graphical circular map of the genomes of strains NKC19-3^T^ (A), NKC19-16^T^ (B), and DSM25609^T^ (C). From outside in: contigs (grey), annotated reference genes (specifically, CDSs) on the forward strand (second circle from outside), and annotated reference genes on the reverse strand (third circle from outside). Different colors were assigned to indicate Cluster of Orthologous Groups of proteins (COG) functions. The fourth circles show rRNA (red) and Trna (blue) genes in both genomes. The inner circle shows GC skew, with green and red indicating positive and negative values, respectively. The innermost circle shows the GC content; blue and deep yellow indicate positive and negative signs, respectively.

Fig. S3. ANI-based phylogenetic tree of the genus *Virgibacillus*. The phylogenetic tree represents the relationships among 28 species within the genus *Virgibacillus* based on average nucleotide identity (ANI) values. The tree was constructed using ANI distances calculated from whole-genome sequences, with branch lengths reflecting genetic distances. Scale bae represents 0.2 indicates the evolutionary distance based on ANI values.

Fig. S4. Core-genome-based phylogenetic tree of the genus *Virgibacillus*. The tree represents the evolutionary relationships among 28 species within the genus *Virgibacillus*, constructed using alignments of core-genome sequences derived from pan-genome analysis. The core-genome consists of highly conserved genes shared across all analyzed genomes, providing a robust framework for resolving phylogenetic relationships. Scale bar 0.1 indicates the number of substitutions per site in the core-genome alignment.

Fig. S5. Two-dimensional thin-layer chromatography (TLC) of polar lipids from strains NKC19-3^T^ (A), NKC19-16^T^ (B), and DSM25609^T^ (C). Spots of total lipids were identified by spraying 5% ethanolic molybdophosphoric acid on the TLC plate. Abbreviations: DPG, diphosphatidylglycerol; PG, phosphatidylglycerol; APL, unidentified aminophospholipid; GL, unidentified glycolipid; PL, unidentified phospholipid; L, unidentified lipid.

## References

[b1-jm-2501001] Amziane M, Metiaz F, Darenfed-Bouanane A, Djenane Z, Selama O (2013). *Virgibacillus natechei* sp. nov., a moderately halophilic bacterium isolated from sediment of a saline lake in southwest of Algeria. Curr Microbiol.

[b2-jm-2501001] An SY, Asahara M, Goto K, Kasai H, Yokota A (2007). *Virgibacillus halophilus* sp. nov., spore-forming bacteria isolated from soil in Japan. Int J Syst Evol Microbiol.

[b3-jm-2501001] Arahal DR, Marquez MC, Volcani BE, Schleifer KH, Ventosa A (1999). *Bacillus marismortui* sp. nov., a new moderately halophilic species from the Dead Sea. Int J Syst Bacteriol.

[b4-jm-2501001] Arahal DR, Marquez MC, Volcani BE, Schleifer KH, Ventosa A (2000). Reclassification of *Bacillus marismortui* as *Salibacillus marismortui* comb. nov. Int J Syst Evol Microbiol.

[b5-jm-2501001] Beck AK, Baker A, Kelly PJ, Deane FP, Shakeshaft A (2016). Protocol for a systematic review of evaluation research for adults who have participated in the 'SMART recovery' mutual support programme. BMJ Open.

[b6-jm-2501001] Cantalapiedra CP, Hernandez-Plaza A, Letunic I, Bork P, Huerta-Cepas J (2021). eggNOG-mapper v2: functional annotation, orthology assignments, and domain prediction at the metagenomic scale. Mol Biol Evol.

[b7-jm-2501001] Carrasco IJ, Marquez CM, Ventosa A (2009). *Virgibacillus salinus* sp. nov., a moderately halophilic bacterium from sediment of a saline lake. Int J Syst Evol Microbiol.

[b8-jm-2501001] Chaudhari N, Gupta V, Dutta C (2016). BPGA- an ultra-fast pan-genome analysis pipeline. Sci Rep.

[b9-jm-2501001] Felsenstein J (1985). Confidence limits on phylogenies: an approach using the bootstrap. Evolution.

[b10-jm-2501001] Fitch WM (1971). Toward defining the course of evolution: minimum change for a specific tree topology. Syst Zool.

[b11-jm-2501001] Galperin MY, Makarova KS, Wolf YI, Koonin EV (2015). Expanded microbial genome coverage and improved protein family annotation in the COG database. Nucleic Acids Res.

[b12-jm-2501001] Guo LY, Wang NN, Wang XQ, Chen GJ, Du ZJ (2017). *Lentibacillus sediminis* sp. nov., isolated from a marine saltern. Int J Syst Evol Microbiol.

[b13-jm-2501001] Heyndrickx M, Lebbe L, Kersters K, De Vos P, Forsyth G (1998). *Virgibacillus*: a new genus to accommodate *Bacillus pantothenticus* (Proom and Knight 1950). Emended description of *Virgibacillus pantothenticus*. Int J Syst Bacteriol.

[b14-jm-2501001] Heyrman J, Logan NA, Busse HJ, Balcaen A, Lebbe L (2003). *Virgibacillus carmonensis* sp. nov., *Virgibacillus necropolis* sp. nov. and *Virgibacillus picturae* sp. nov., three novel species isolated from deteriorated mural paintings, transfer of the species of the genus *Salibacillus* to *Virgibacillus*, as *Virgibacillus marismortui* comb. nov. and *Virgibacillus salexigens* comb. nov., and emended description of the genus *Virgibacillus*. Int J Syst Evol Microbiol.

[b15-jm-2501001] Hiraishi A, Ueda Y, Ishihara J (1998). Quinone profiling of bacterial communities in natural and synthetic sewage activated sludge for enhanced phosphate removal. Appl Environ Microbiol.

[b16-jm-2501001] Huerta-Cepas J, Forslund K, Coelho LP, Szkarczyk D, Jensen LJ (2017). Fast genome-wide functional annotation through orthology assignment by eggNOG-Mapper. Mol Biol Evol.

[b17-jm-2501001] Kim J, Jung MJ, Roh SW, Nam YD, Shin KS (2011). *Virgibacillus alimentarius* sp. nov., isolated from a traditional Korean food. Int J Syst Evol Microbiol.

[b18-jm-2501001] Kimura M (1980). A simple method for estimating evolutionary rates of base substitutions through comparative studies of nucleotide sequences. J Mol Evol.

[b19-jm-2501001] Kumar S, Stecher G, Li M, Knyaz C, Tamura K (2018). MEGAX: Molecular Evolutionary Genetics Analysis across computing platforms. Mol Biol Evol.

[b20-jm-2501001] Lane DJ (1991).

[b21-jm-2501001] Lovley DR, Phillips EJ (1988). Novel mode of microbial energy metabolism: Organic carbon oxidation coupled to dissimilatory reduction of iron or manganese. Appl Environ Microbiol.

[b22-jm-2501001] Nei M, Kumar S (2000).

[b23-jm-2501001] Oh YJ, Jang JY, Lim SK, Kwon MS, Lee J (2017). *Virgibacillus kimchii* sp. nov., a halophilic bacterium isolated from kimchi. J Microbiol.

[b24-jm-2501001] Oh YJ, Lee HW, Lim SK, Kwon MS, Lee J (2016). *Gracilibacillus kimchii* sp. nov., a halophilic bacterium isolated from kimchi. J Microbiol.

[b25-jm-2501001] Pritchard L, Glover RH, Humphris S, Elphinstone JG, Toth IK (2016). Genomics and taxonomy in diagnostics for food security: soft-rotting enterobacterial plant pathogens. Anal Methods.

[b26-jm-2501001] Rzhetsky A, Nei M (1992). A simple method for estimating and testing minimum evolution trees. Mol Biol Evol.

[b27-jm-2501001] Saitou N, Nei M (1987). Neighbor-joining method: a new method for reconstructing phylogenetic trees. Mol Biol Evol.

[b28-jm-2501001] Salzman AT, Fulton S, Gordon N, Meys M, Carberry R (1993). Development and execution of a biomolecule purification method. Am Biotechnol Lab.

[b29-jm-2501001] Sasser M (1990).

[b30-jm-2501001] Seiler H, Wenning M (2013). *Virgibacillus halotolerans* sp. nov., isolated from a dairy product. Int J Syst Evol Microbiol.

[b31-jm-2501001] Tak EJ, Kim HS, Lee JY, Kang W, Sung H (2018). *Virgibacillus phasianinus* sp. nov., a halophilic bacterium isolated from faeces of a Swinhoe’s pheasant, *Lophura swinhoii*. Int J Syst Evol Microbiol.

[b32-jm-2501001] Tatusova T, DiCuccio M, Badretdin A, Chetvernin V, Nawrocki EP (2016). NCBI prokaryotic genome annotation pipeline. Nucleic Acids Res.

[b33-jm-2501001] Tindall BJ, Tomlinson GA, Hochstein LI (1987). Polar lipid composition of a new halobacterium. Syst Appl Microbiol.

[b34-jm-2501001] Tittsler RP, Sandholzer LA (1936). The use of semi-solid agar for the detection of bacterial motility. J Bacteriol.

[b35-jm-2501001] Wang XW, Xue YF, Ma YH (2010). *Virgibacillus subterraneus* sp. nov., a moderately halophilic Gram-positive bacterium isolated from subsurface saline soil. Int J Syst Evol Microbiol.

[b36-jm-2501001] Xu B, Hu B, Wang J, Lan Y, Zhu Y (2018). *Virgibacillus indicus* sp. nov. and *Virgibacillus profundi* sp. nov., two moderately halophilic bacteria isolated from marine sediment by using microfluidic streak plates. Int J Syst Evol Microbiol.

[b37-jm-2501001] Yin X, Yang Y, Wang S, Zhang G (2015). *Virgibacillus oceani* sp. nov. isolated from ocean sediment. Int J Syst Evol Microbiol.

[b38-jm-2501001] Yoon SH, Ha SM, Kwon S, Lim J, Kim Y (2017). Introducing EzBioCloud: a taxonomically united database of 16S rRNA gene sequences and whole-genome assemblies. Int J Syst Evol Microbiol.

[b39-jm-2501001] Yoon JH, Kang SJ, Jung YT, Lee KC, Oh HW (2010). *Virgibacillus byunsanensis* sp. nov., isolated from a marine solar saltern. Int J Syst Evol Microbiol.

[b40-jm-2501001] Yoon JH, Kang KH, Park YH (2002). *Lentibacillus salicampi* gen. nov., sp. nov., a moderately halophilic bacterium isolated from a salt field in Korea. Int J Syst Evol Microbiol.

[b41-jm-2501001] Yoon JH, Oh TK, Park YH (2004). Transfer of *Bacillus halodenitrificans* Denariaz et al. 1989 to the genus *Virgibacillus* as *Virgibacillus halodenitrificans* comb. nov. Int J Syst Evol Microbiol.

[b42-jm-2501001] Zhang YJ, Zhou Y, Ja M, Shi R, Chun-Yu WX (2012). *Virgibacillus albus* sp. nov., a novel moderately halophilic bacterium isolated from Lop Nur salt lake in Xinjiang province, China. Antonie van Leeuwenhoek.

