# Supplementary material for: Virgibacillus saliphilus sp. nov. and Virgibacillus salidurans sp. nov., isolated from kimchi
Source: J Microbiol. 2025 Jan 24;63(1):e.2501001. doi: 10.71150/jm.2501001 (PMC13577018; doi:10.71150/jm.2501001)
Supplement: Supplementary file 1 [file jm-2501001-Supplementary-Fig-S1.pdf]

Supplementary Figures and Tables

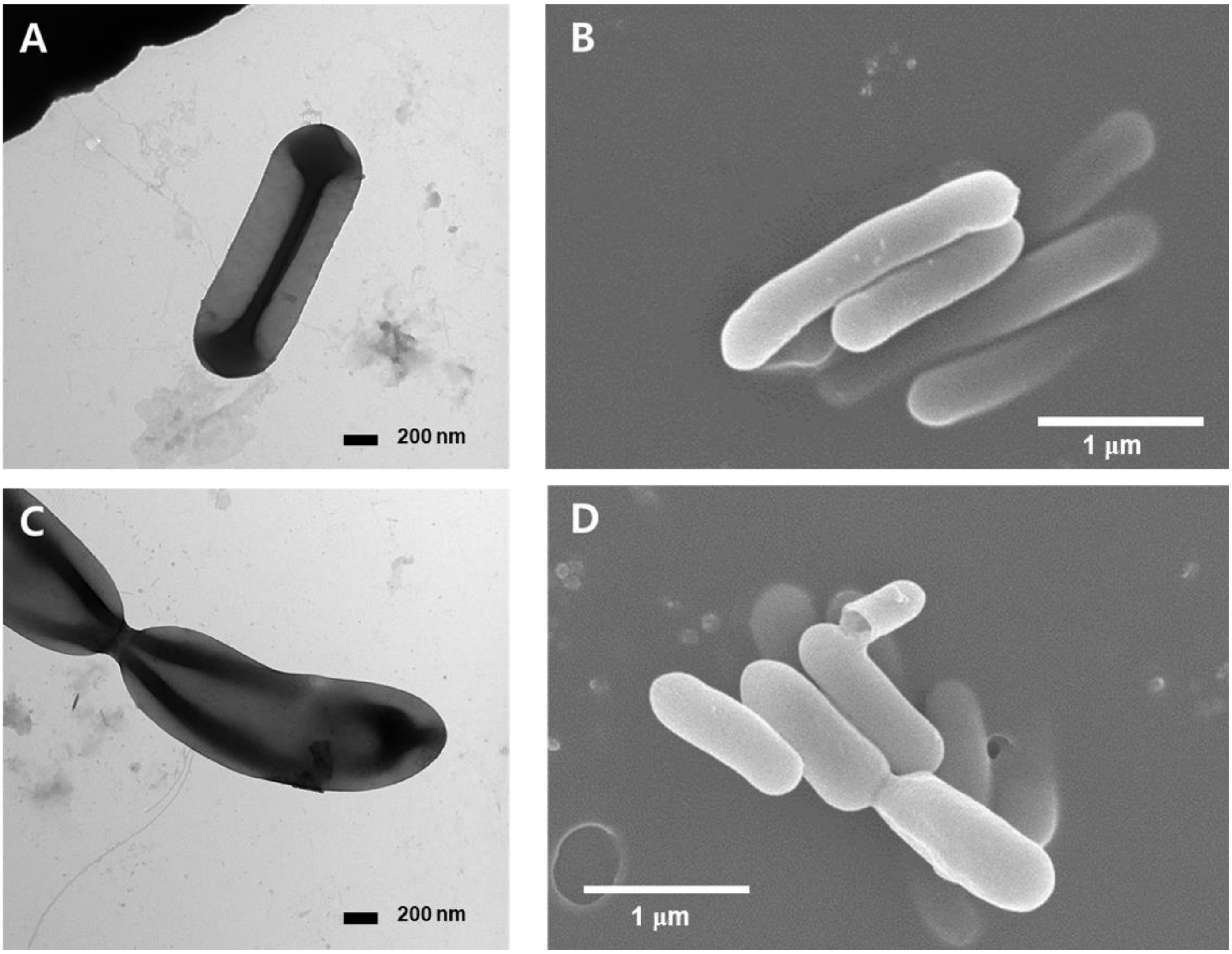

Fig. S1. Transmission electron micrographs and scanning electron micrographs of strains NKC19-3^T (A, B) and NKC19-16^T (C, D) grown in M1730 medium supplemented with 10% NaCl for 5 days.
