# Supplementary material for: Virgibacillus saliphilus sp. nov. and Virgibacillus salidurans sp. nov., isolated from kimchi
Source: J Microbiol. 2025 Jan 24;63(1):e.2501001. doi: 10.71150/jm.2501001 (PMC13577018; doi:10.71150/jm.2501001)
Supplement: Supplementary file 2 [file jm-2501001-Supplementary-Fig-S2.pdf]

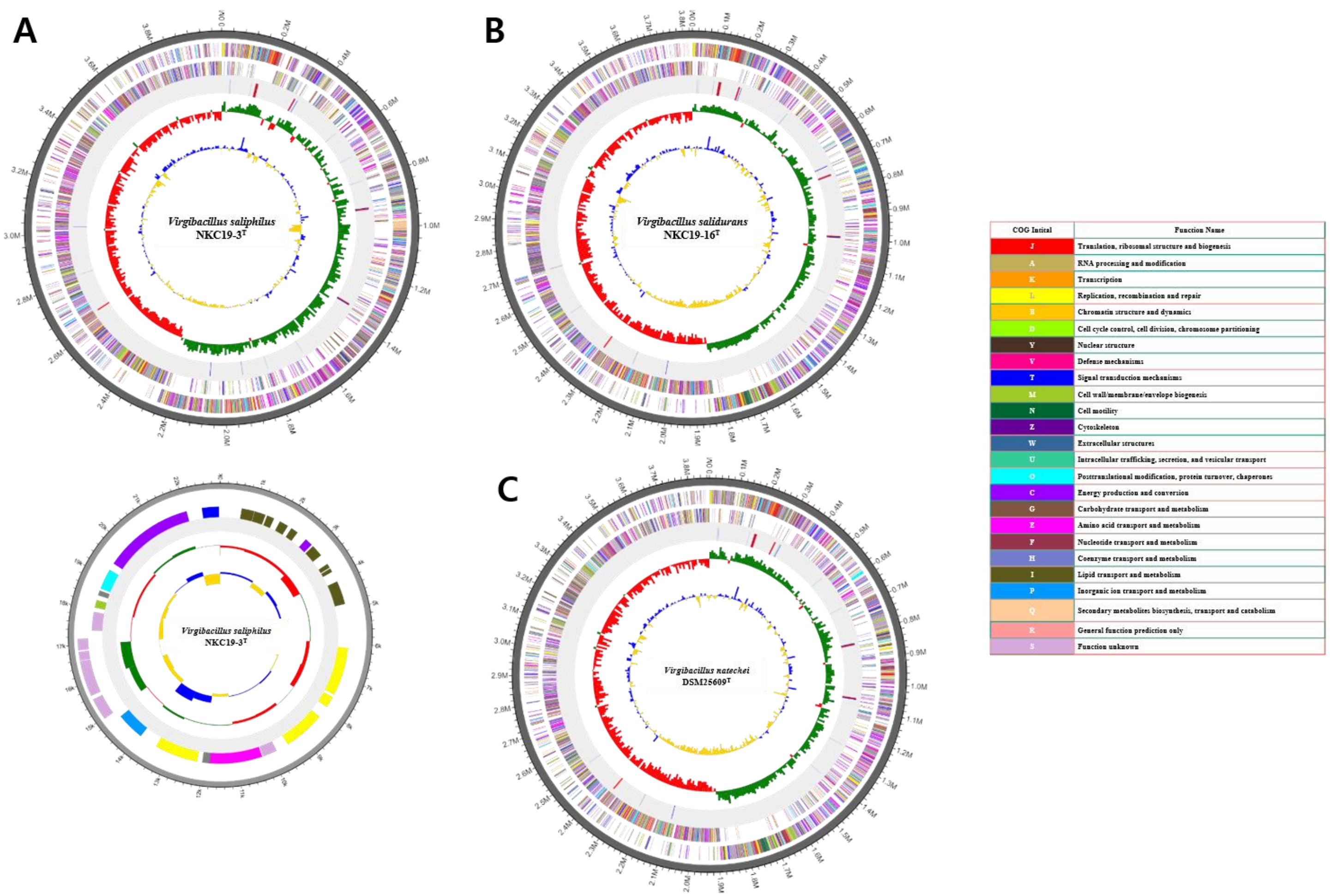

Fig. S2. Graphical circular map of the genomes of strains NKC19-3^{T} (A), NKC19-16^{T} (B), and DSM25609^{T} (C). From outside in: contigs (grey), annotated reference genes (specifically, CDSs) on the forward strand (second circle from outside), and annotated reference genes on the reverse strand (third circle from outside). Different colors were assigned to indicate Cluster of Orthologous Groups of proteins (COG) functions. The fourth circles show rRNA (red) and tRNA (blue) genes in both genomes. The inner circle shows GC skew, with green and red indicating positive and negative values, respectively. The innermost circle shows the GC content; blue and deep yellow indicate positive and negative signs, respectively.
