# Supplementary material for: Virgibacillus saliphilus sp. nov. and Virgibacillus salidurans sp. nov., isolated from kimchi
Source: J Microbiol. 2025 Jan 24;63(1):e.2501001. doi: 10.71150/jm.2501001 (PMC13577018; doi:10.71150/jm.2501001)
Supplement: Supplementary file 3 [file jm-2501001-Supplementary-Fig-S3.pdf]

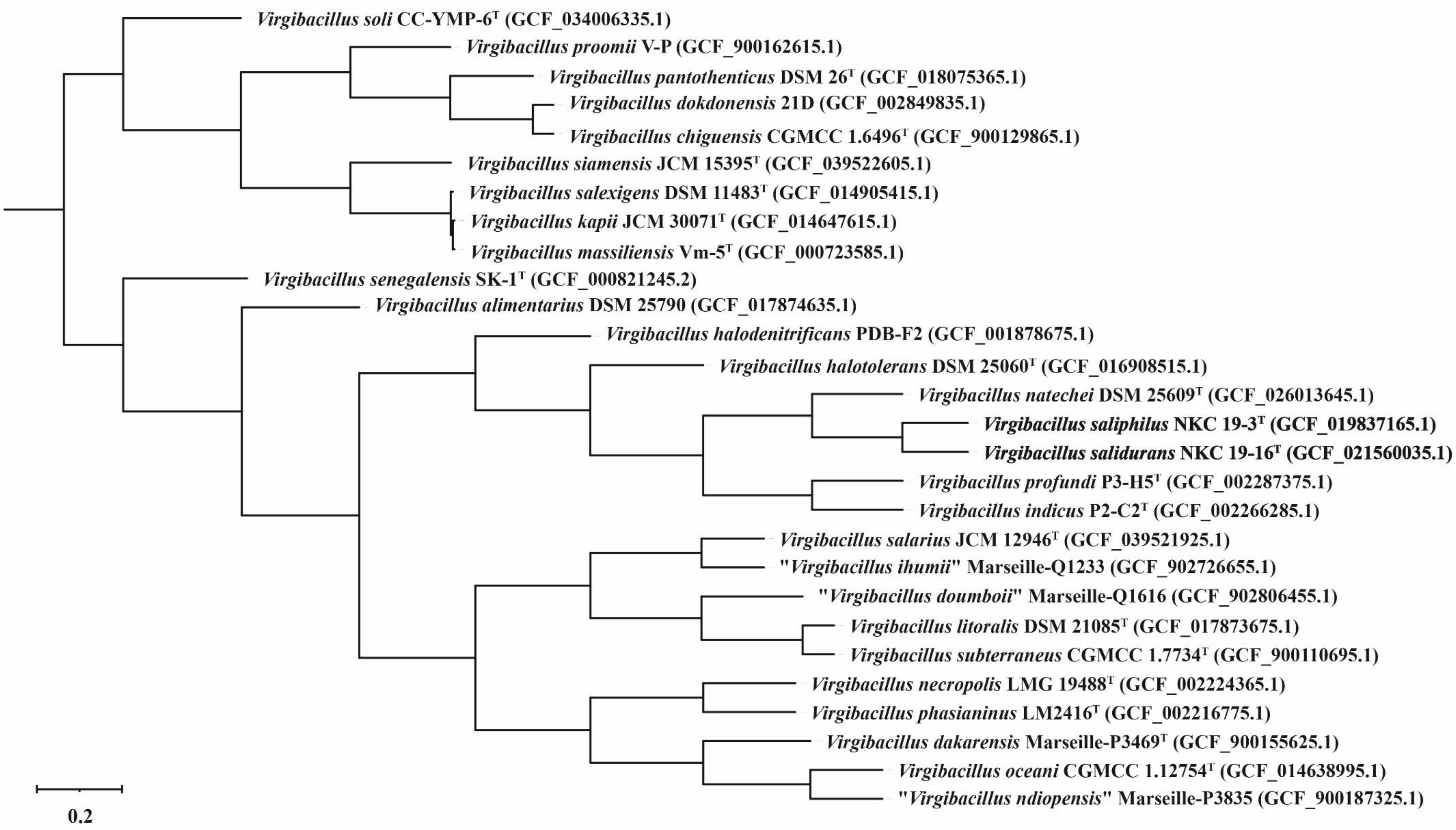


**Fig. S3.** ANI-based phylogenetic tree of the genus *Virgibacillus.* The phylogenetic tree represents the relationships among 28 species within the genus *Virgibacillus* based on average nucleotide identity (ANI) values. The tree was constructed using ANI distances calculated from whole-genome sequences, with branch lengths reflecting genetic distances. Scale bae represents 0.2 indicates the evolutionary distance based on ANI values.
