# Supplementary material for: Virgibacillus saliphilus sp. nov. and Virgibacillus salidurans sp. nov., isolated from kimchi
Source: J Microbiol. 2025 Jan 24;63(1):e.2501001. doi: 10.71150/jm.2501001 (PMC13577018; doi:10.71150/jm.2501001)
Supplement: Supplementary file 4 [file jm-2501001-Supplementary-Fig-S4.pdf]

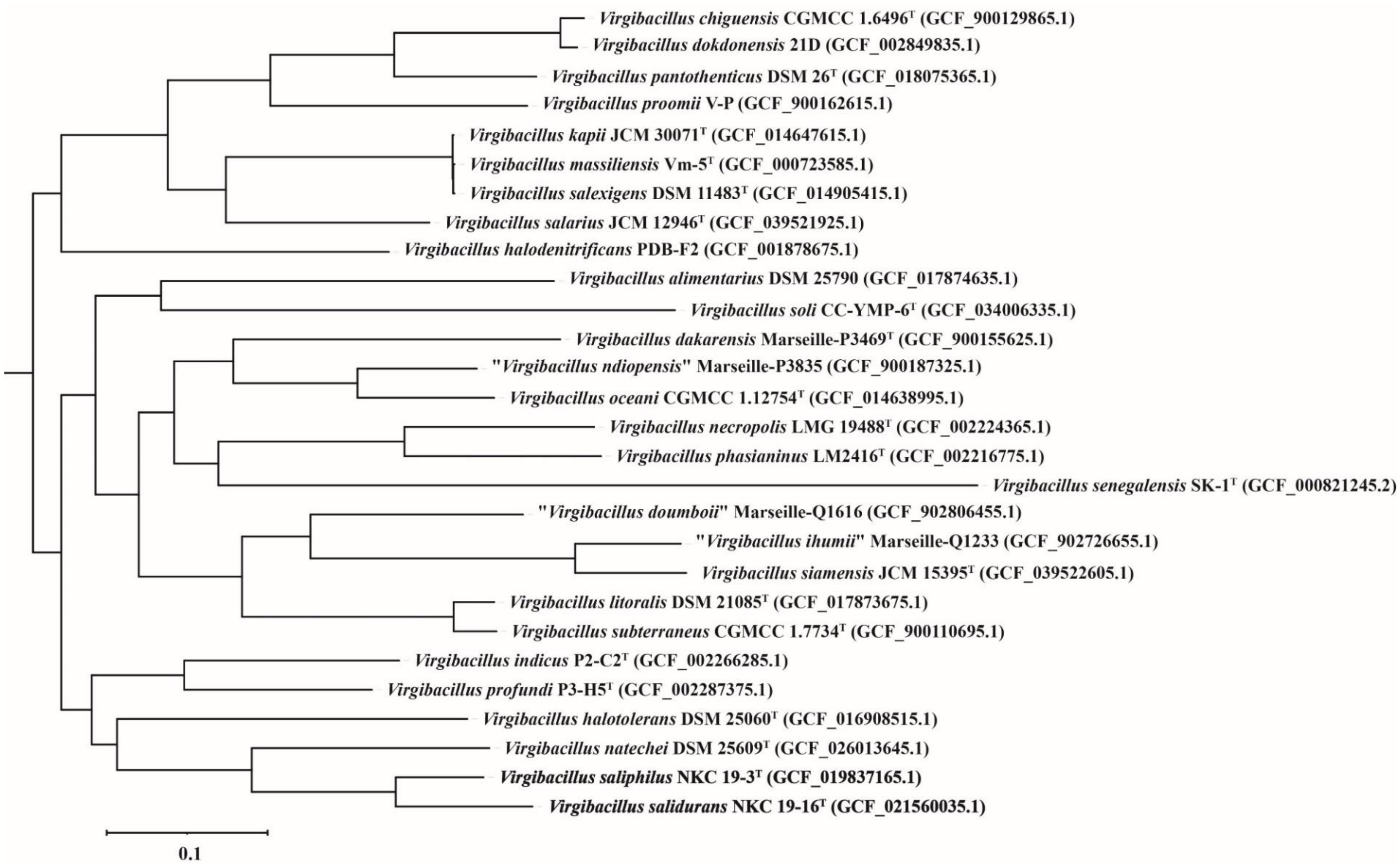

Fig. S4. Core-genome-based phylogenetic tree of the genus *Virgibacillus*. The tree represents the evolutionary relationships among 28 species within the genus Virgibacillus, constructed using alignments of core-genome sequences derived from pan-genome analysis. The core-genome consists of highly conserved genes shared across all analyzed genomes, providing a robust framework for resolving phylogenetic relationships. Scale bar 0.1 indicates the number of substitutions per site in the core-genome alignment.
