# Supplementary material for: Virgibacillus saliphilus sp. nov. and Virgibacillus salidurans sp. nov., isolated from kimchi
Source: J Microbiol. 2025 Jan 24;63(1):e.2501001. doi: 10.71150/jm.2501001 (PMC13577018; doi:10.71150/jm.2501001)
Supplement: Supplementary file 5 [file jm-2501001-Supplementary-Fig-S5.pdf]

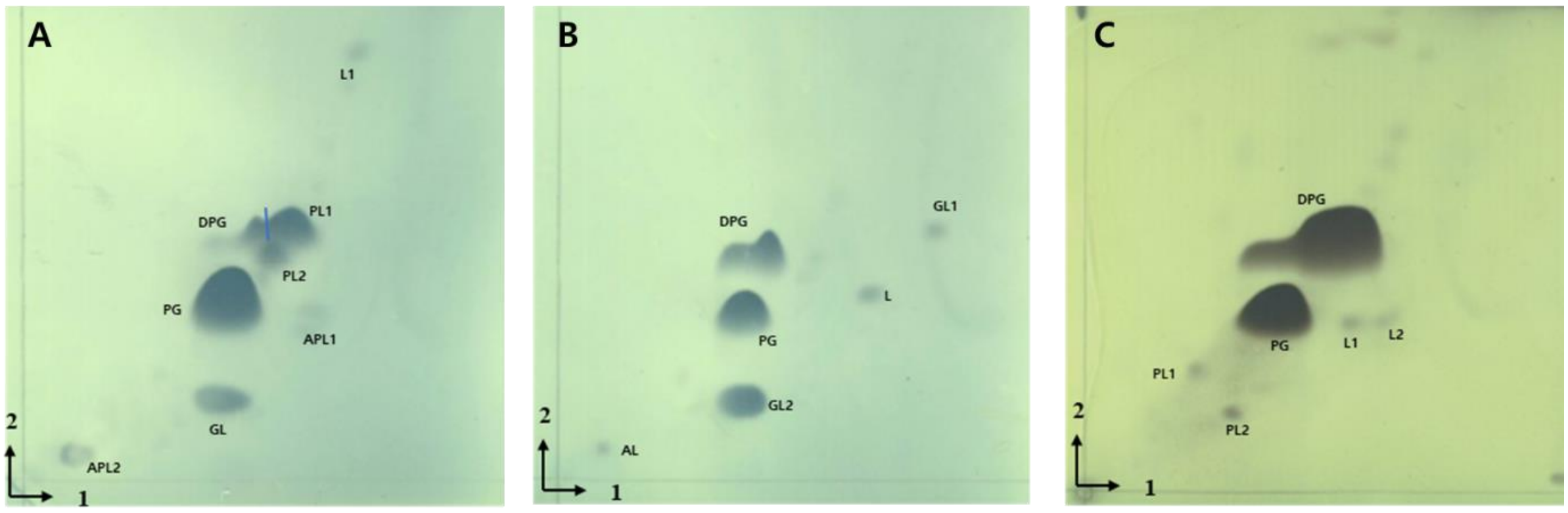

Fig. S5. Two-dimensional thin-layer chromatography (TLC) of polar lipids from strains NKC19-3^T (A), NKC19-16^T (B), and DSM25609^T (C). Spots of total lipids were identified by spraying 5% ethanolic molybdophosphoric acid on the TLC plate. Abbreviations: DPG, diphosphatidylglycerol; PG, phosphatidylglycerol; APL, unidentified aminophospholipid; GL, unidentified glycolipid; PL, unidentified phospholipid; L, unidentified lipid.
