# Supplementary material for: Virgibacillus saliphilus sp. nov. and Virgibacillus salidurans sp. nov., isolated from kimchi
Source: J Microbiol. 2025 Jan 24;63(1):e.2501001. doi: 10.71150/jm.2501001 (PMC13577018; doi:10.71150/jm.2501001)
Supplement: Supplementary file 6 [file jm-2501001-Supplementary-Table-S1.pdf]

**Table S1.** Genomic features of strains NKC19-3$^T$, NKC19-16$^T$, and DSM25609$^T$. All data were from this study unless stated otherwise.

Strains: 1, NKC19-3$^T$; 2, NKC19-16$^T$; 3, *Virgibacillus natechei* DSM25609$^T$.

| **Properties** | **1** | **2** | **3** |
|---|---|---|---|
| BioProject | PRJNA726327 | PRJNA726329 | PRJNA894318 |
| BioSample | SAMN18927773 | SAMN18927775 | SAMN31452450 |
| Accession No. | JAGYHC000000000 | CP074373 | CP110224 |
| Sequencing method | PacBio RSII | PacBio RSII | PacBio RSII |
| Assembly method | HGAP v. 3.0 | HGAP v. 3.0 | HGAP v. 3.0 |
| Genome size (bp) | 4,054,365 | 3,829,764 | 3,867,590 |
| Contig | 2 | 1 | 1 |
| Genome coverage | 122.3x | 343.7x | 322.6x |
| CDSs (total) | 3,785 | 3,726 | 3,776 |
| rRNA genes | 88 | 86 | 85 |
| tRNA genes | 65 | 63 | 62 |
| G + C content (mol%) | 38.9 | 39.5 | 38.4 |

CDSs, coding sequence
